# Factors Associated with Usage of Oral-PrEP among Female Sex Workers in Nairobi, Kenya, Assessed by Self-Report and a Point-of-Care Urine Tenofovir Immunoassay

**DOI:** 10.1007/s10461-024-04455-3

**Published:** 2024-08-13

**Authors:** Pooja Shah, Matthew Spinelli, Erastus Irungu, Rhoda Kabuti, Pauline Ngurukiri, Hellen Babu, Mary Kungu, The Maisha Fiti Study Champions, Chrispo Nyabuto, Anne Mahero, Karen Devries, Nambusi Kyegombe, Graham F. Medley, Mitzy Gafos, Janet Seeley, Helen A. Weiss, Rupert Kaul, Monica Gandhi, Tara S. Beattie, Joshua Kimani

**Affiliations:** 1 Department of Global Health and Development, Faculty of Public Health and Policy, London School of Hygiene and Tropical Medicine, Keppel Street, London, WC1E 7HT UK; 2 Division of HIV, Infectious Diseases, and Global Medicine, University of California, San Francisco, San Francisco, CA USA; 3 Partners for Health and Development in Africa (PHDA), Nairobi, Kenya; 4 Present Address: MRC/UVRI and LSHTM Uganda Research Unit, Entebbe, Uganda; 5 MRC International Statistics & Epidemiology Group, Department of Infectious Disease Epidemiology and International Health, London School of Hygiene and Tropical Medicine, London, UK; 6 Departments of Immunology and Medicine, University of Toronto, Toronto, Canada; 7 Africa Health Research Institute, Durban, Kwa-Zulu Natal South Africa

**Keywords:** Female sex workers, Kenya, Hierarchical modelling, HIV prevention, PrEP, Adolescent girls and young women

## Abstract

Pre-exposure prophylaxis (PrEP) is highly effective at reducing HIV acquisition. We aimed to estimate usage of oral-PrEP, and factors associated with adherence among female sex workers (FSWs) in Nairobi, Kenya, using a novel point-of-care urine tenofovir lateral flow assay (LFA). The Maisha Fiti study randomly selected FSWs from Sex Worker Outreach Program clinics in Nairobi. Data were collected from 1003 FSWs from June-October 2019, including surveys on self-reported oral-PrEP adherence. Adherence was also measured using the LFA for HIV-negative FSWs currently taking oral-PrEP. Informed by a social-ecological theoretical framework, we used hierarchical multivariable logistic regression models to estimate associations between individual, interpersonal/community, and structural/institutional-level factors and either self-reported or LFA-assessed adherence. Overall, 746 HIV-negative FSWs aged 18–40 participated in the study, of whom 180 (24.1%) self-reported currently taking oral-PrEP. Of these, 56 (31.1%) were adherent to oral-PrEP as measured by LFA. In the multivariable analyses, associations with currently taking oral-PrEP included having completed secondary education, high alcohol/substance use, feeling empowered to use PrEP, current intimate partner, no recent intimate partner violence, having support from sex worker organisations, experiencing sex work-related stigma, and seeking healthcare services despite stigma. Associations with oral-PrEP LFA-measured adherence measured included having only primary education, experience of childhood emotional violence, belonging to a higher wealth tertile, and being nulliparous. Oral-PrEP adherence, measured by self-report or objectively, is low among FSWs in Nairobi. Programs to improve oral-PrEP usage among FSWs should work to mitigate social and structural barriers and involve collaboration between FSWs, healthcare providers and policymakers.

## Introduction

Women who engage in sex work have a high burden of HIV due to a complex intersection of structural, social, and economic factors [1]. Female sex workers (FSWs) are defined as women who receive goods or money in exchange for sex, either occasionally or regularly, with explicit acknowledgment and without any mention of a normatively recognised relationship [2, 3]. FSWs have a 13 times higher risk of acquiring HIV than adult women in the general population [4], and a 2013 study estimated that female sex work without condom use contributed to approximately 15% of all HIV infections among women globally [5]. This risk is augmented by the challenges faced by FSWs on a daily basis, including criminalisation of sex work, discrimination, economic hardship, difficulty negotiating condom use, and high levels of physical and/or sexual violence from clients, intimate partners, and other perpetrators [4, 6–9].

Correctly and consistently taking a daily oral regimen containing tenofovir-based medications for oral pre-exposure prophylaxis (oral-PrEP) reduces the risk of HIV acquisition through sex by 99% or higher [10, 11]. Modelling studies from sub-Saharan Africa (SSA) have shown that effective oral-PrEP usage among FSWs would result in the greatest HIV incidence reduction at a population level, of all populations at high risk of HIV infection [12, 13]. However, oral-PrEP’s efficacy is dependent on adequate adherence at times of potential sexual exposure [14]. In 2017, Kenya was one of the first African countries to implement a national programme of oral-PrEP provision for key populations, mainly through community-led drop-in healthcare centres [15].

Research among key populations – including FSWs in SSA – has found high levels of awareness of and acceptability to oral-PrEP [16–18], yet levels of uptake and adherence remain suboptimal, and discontinuation is frequent [19]. Adherence to oral-PrEP is complex and measurement is challenging. Current methods predominantly measure continuous adherence, which does not account for the variable risk of HIV acquisition faced by FSWs because of their occupation. ‘Prevention-effective adherence’ is a concept that acknowledges this dynamic risk and concurrent use of other HIV prevention methods and should be considered by HIV prevention programs to enhance the measurement of oral-PrEP adherence [20].

Self-reported adherence is the most common method to assess adherence, due to convenience and feasibility, but can be plagued by social desirability and recall biases. Commonly implemented objective measures of adherence include measuring antiretroviral drug levels in biological samples of plasma [21] or dried blood spots (DBS) [22] using spectrometry-based technology. These direct objective measures are reliable but are expensive, require skilled laboratory personnel and blood draws, and are mainly used in research rather than care settings [23]. Indirect objective methods such as pill count are less expensive and do not require specialised laboratory devices to analyse but are labour-intensive [24]. Pill counts and self-reporting (both of which are inexpensive and easy to perform) are both commonly used to measure adherence in HIV prevention and treatment care settings in SSA, and studies have reported a strong correlation [25–27] and moderate to high agreement [27–29] between the two methods. However, both methods have their limitations, including overestimation, reporting bias, and recall errors [28, 30].

A recent advance in oral-PrEP adherence measurement is the development of urine-based lateral flow assays (LFAs) that provide objective adherence information to tenofovir-based PrEP in 2–3 min. LFAs, the most common example of which are urine pregnancy tests, are affordable and can be used at the point of care. The validated LFA test used in this study is highly specific and sensitive when compared to the gold-standard method of liquid chromatography-tandem mass spectrometry [31], and can accurately determine dosing over the past four to seven days [31–33].

Studies on the effectiveness of oral-PrEP after its approval among adolescent girls and young women (AGYW) in SSA have presented mixed results. Data from some national oral-PrEP programs in Kenya, Zimbabwe, and Zambia showed moderate levels of uptake (20–35%) with poor adherence [34–37]. These studies highlight the need to better understand structural, social, and behavioural factors influencing oral-PrEP use [38–40]. FSWs in SSA face structural and social challenges such as poverty, stigma, and gender-based violence, as well as varying patterns and locations of work [1], resulting in barriers to effective oral-PrEP uptake and adherence [41].

This study aimed to assess oral-PrEP adherence among FSWs who were HIV-negative in Nairobi, Kenya, and to use a social-ecological model to evaluate factors associated with oral-PrEP self-reported adherence and LFA-measured adherence using the novel urine-based LFA.

## Methods

### Study Setting

Within Nairobi (a city of 4.4 million) [42], an estimated 40,000 FSWs operate from approximately 2,000 different known locations and elsewhere [43, 44]. The Sex Workers Outreach Program (SWOP) was established in 2008 through a collaboration with Partners for Health and Development in Africa (PHDA) and the University of Manitoba, with funding provided by the President’s Emergency Plan for AIDS Relief (PEPFAR). SWOP provides free clinical care, harm-reduction measures, and counselling support through seven clinics to ‘key populations’ including approximately 33,000 FSWs across Nairobi.

### Study Population and Procedures

Maisha Fiti was a mixed-methods longitudinal study that explored the impact of social and structural factors on immunological changes in the blood and genital tract of FSWs. The Maisha Fiti study design, procedures, and data collection have been described in detail elsewhere [45, 46]. In brief, the study was designed to have enough power to identify genital inflammation in women with recent experience of sexual or physical violence.

Eligibility criteria for Maisha Fiti included women who self-identified as currently active sex workers, had visited a SWOP clinic in the past 12 months, were not currently pregnant or breastfeeding, and did not suffer from any chronic illnesses other than HIV. The sampling frame comprised 10,292 FSWs, of whom 1,200 FSWs were randomly selected, in proportion to the size of each SWOP clinic. Potential participants were provided information over the phone, and those interested were given detailed study information and screened for eligibility at a dedicated study clinic. After providing written informed consent, at the baseline study visit, participants completed a behavioural-biological survey and were provided with clinical and psychological support. Participants were reimbursed 500 KSH (~ 5 USD) for their time and travel costs.

### Ethics Statement

Institutional ethics approval for this study was obtained from Kenyatta National Hospital and University of Nairobi Research Ethics Committee (KNH ERC P778/11/2018), the London School of Hygiene and Tropical Medicine Research Ethics Committee (Approval number: 16229), and the University of Toronto Research Ethics Board (Approval number: 37046).

### Data Collection

At baseline, participants completed a behavioural-biological survey administered by a trained researcher in either Swahili or English based on preference. All consenting participants also provided blood and urine samples, collected during the baseline visit and frozen for future testing.

### Outcome Variables

Among those who were HIV-negative, self-reported current usage of oral-PrEP at baseline was estimated using the question “*Are you currently taking PrEP?*” with responses recorded as ‘yes/no’.

Among FSWs who reported currently taking oral-PrEP, adherence to oral-PrEP in the past week was estimated using two methods:

- Self-reported oral-PrEP adherence was estimated using the question, “*Have you missed any PrEP doses in the last seven days?*” and those answering ‘no’ were categorised as being adherent to oral-PrEP based on self-report.
- Objective oral-PrEP adherence was estimated using an LFA urine test.

### Laboratory Procedures

HIV status was screened for using rapid HIV tests. Blood samples were used to confirm positive HIV tests using HIV DNA Genexpert. Frozen urine samples were used to test for oral-PrEP adherence using the previously described LFA (Abbott Rapid Diagnostics Division). The LFA uses a tenofovir detection threshold of 1,500 ng/mL, which has 99% sensitivity and 97% specificity for detecting tenofovir in urine 24 h after a dose of PrEP is taken, and high (86%) sensitivity for nonadherence at 96 h [31]. It has been tested in diverse populations and when tested among heterosexual men and women in Kenya, was shown to be over 98% sensitive and specific [47].

### Conceptual Framework

**Fig. 1:**
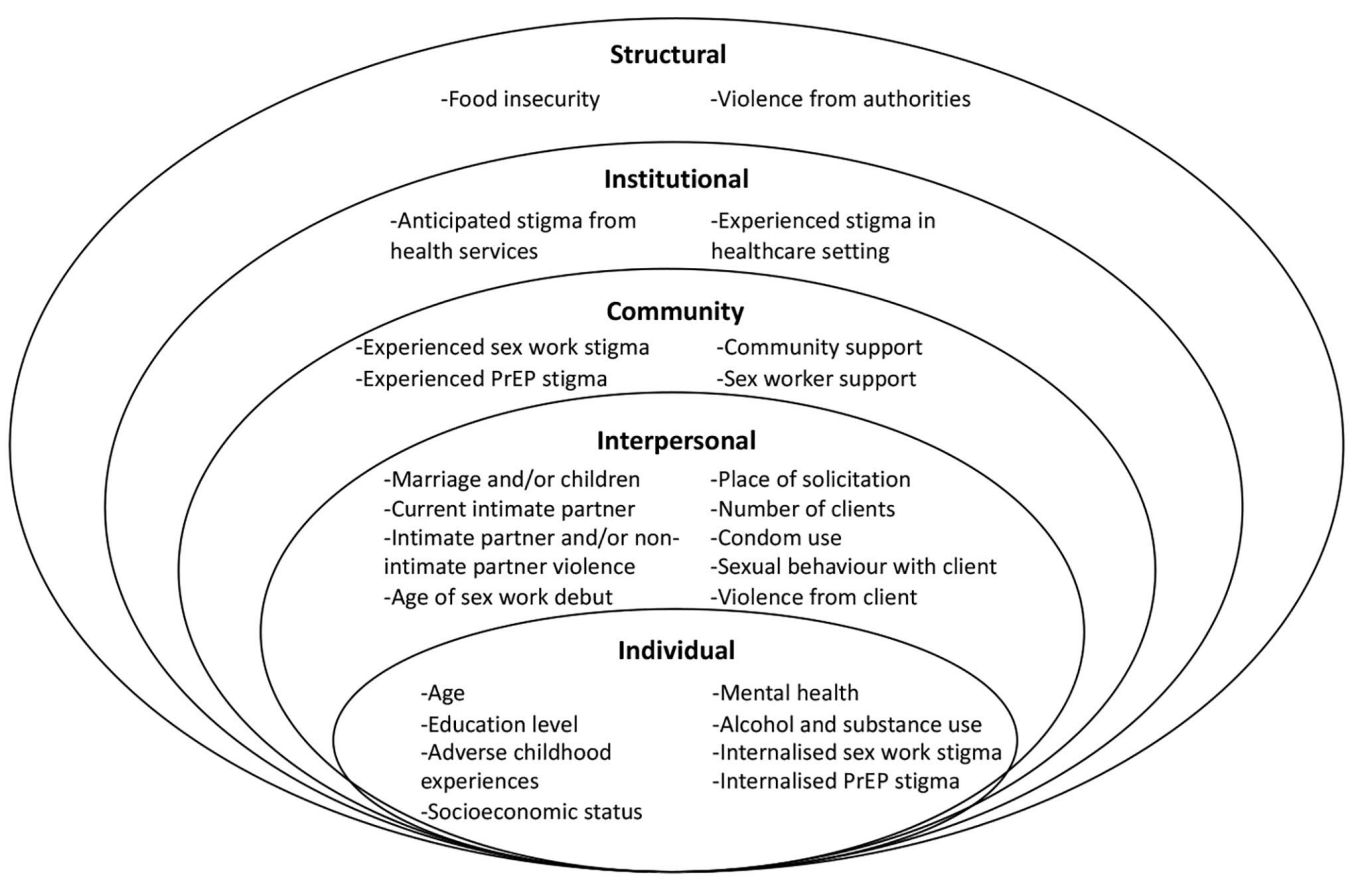
Social-ecological framework of factors influencing PrEP usage among female sex workers

Drawing on previous research [48–50] and preliminary data, we developed a conceptual framework (Fig. 1) based on the social-ecological model to analyse associations between structural and social factors and PrEP usage. This model contextualises how factors at five interdependent levels (individual, interpersonal, community, organisational, and structural) influence health behaviours [51, 52]. Factors at the individual level included socio-demographic factors, adverse childhood experiences, mental health problems (depression, anxiety, PTSD), alcohol and substance use, and internalised stigma. Interpersonal-level factors included intimate relationships and sex work clients, and community-level factors included experiencing stigma and support. Institutional-level factors included healthcare-related stigma, and structural factors included food insecurity and violence from authorities. Details on hypothesised exposure variables, including validated measures used, are provided in Table 1. For the statistical analysis, variables were grouped into levels. Level 1 comprised of sociodemographic characteristics and individual-level variables. Level 2 comprised of interpersonal- and community-level variables. Level 3 comprised of institutional- and structural-level variables.

**Table 1:** Definition of exposure variables

Variable	Measure/Question	Category
Level 1: Sociodemographic characteristics and individual-level factors		
Adverse childhood experiences	World Health Organization (WHO) Adverse Childhood Experiences International Questionnaire (ACE-IQ) [53] During your first 18 years, did you ever live on the street?	No vs. Yes
Wealth tertile	14 questions about household assets as used in the Kenya Demographic and Health Survey 2014 [54]	Household wealth tertiles by principal component analysis: Low; Middle; Upper
Depression and/or anxiety	Patient Health Questionnaire – 9 (PHQ-9) [55, 56] Generalised Anxiety Disorder – 7 Assessment [55, 56]	None 0–4; Mild 5–9; Moderate/severe ≥ 10
Suicidal thoughts/ behaviours	Have you thought about ending your life in the past 30 days? Have you tried to end your life (kill yourself) in the past 30 days?	No vs. Yes
Post-traumatic stress disorder	Harvard Trauma Questionnaire – 17 [57]	Negative 0–2; Positive/at risk ≥ 2
Harmful alcohol and/or substance use	WHO ASSIST (Alcohol, Smoking and Substance Involvement Screening Test) questionnaire [58]	Low risk 0–10; Moderate risk 11–26; High risk ≥ 27
Internalised sex work stigma	I have lost respect or standing in the community because I sell sex I think less of myself because I sell sex I have felt ashamed because I sell sex	No - all answered No; Yes – any question answered yes
Internalised pre-exposure prophylaxis (PrEP) stigma	I feel ashamed of using PrEP I would feel embarrassed if others knew I was using PrEP I feel empowered to use PrEP I think I am not following the ‘rules’ of my community if I take PrEP	No vs. Yes
Level 2: Interpersonal and community level factors		
Age when first started selling sex	How old were you when you first received money/goods in exchange for sex?	≤ 15; 16–17; 18 years
Place of selling sex	Where do you usually meet/pick up/solicit your male clients? Street/bus/taxis – public places Other – Home, sex den/brothel, bar/club/restaurant, lodge/hotel, escort services, massage parlor, markets, phone/mobile, social gatherings, through middleman, internet	No vs. Yes
Experienced sex work stigma	People have talked badly about me because I sell sex I have been insulted/harassed/threatened because I sell sex I have felt excluded from/rejected by my family because I sell sex	No - all answered No; Yes – any question answered yes
Experienced PrEP stigma	I think people will give me a hard time (such as make fun of me or talk badly about me) if I tell them I am taking PrEP I think people will judge me negatively if I take PrEP I think I am at greater risk for physical violence/rape if I take PrEP People will think I am behaving responsibly if I take PrEP	No vs. Yes
Has support from the community	Do you have someone you can talk to about your problems? Do you have any family you can ask for emotional support from? Can you seek emotional support from a religious place of worship?	No - all answered No; Yes – any question answered yes
Has support from sex worker organisations	Are you a member of a sex worker community-based organisation? During the past 12 months, have you ever participated in any activities organised by the national female sex worker networks?	No - all answered No; Yes – any question answered yes
Level 3: Institutional and structural level factors		
Healthcare-related stigma	I have avoided seeking health services because I am worried someone may learn I sell sex I have been denied health services or experienced an increase in cost because I sell sex	No vs. Yes

### Statistical Analysis

Statistical analyses were conducted in STATA version 17.0 (Stata Inc., College Station, TX, USA). A receiver operating characteristic (ROC) curve analysis was used to examine the performance of self-reported oral-PrEP adherence using LFA as the gold standard, utilising ROC curves and the area under the ROC curve (AUROC) [59]. Kappa statistics, sensitivity, and specificity were calculated to compare self-reported adherence to the LFA test [60]. Accuracy was estimated as the number of participants adherent or non-adherent to oral-PrEP as assessed by LFA.

The conceptual framework was used to build multivariable models for each outcome (self-reported oral-PrEP adherence, and adherence assessed by LFA) [61]. Odds ratios (ORs) and confidence intervals (CIs) were used to estimate associations, and *p*-values were calculated by joint hypothesis using the adjusted Wald test (allowing for sampling weights). Tests for trend were carried out for all ordered categorical variables included in the final models. All models were adjusted for participants’ current age and SWOP clinic as fixed a priori defined variables.

The initial model was created by including all Level 1 variables. The variable with the greatest *p*-value was removed before re-estimating, and this process was continued until no variables with *p* > 0.1 remained. Model 2 was created by adding all Level 2 variables to the finalised Model 1 and repeating the process. Model 3 contained Level 1 and 2 variables, and Level 3 variables with *p*-values < 0.1. Missing data were reported if more than 5% of observations were missing.

## Results

### Demographic and Sexual Behaviour Characteristics

Of 1200 sampled women, 1039 were eligible and 1003 (96.5%) provided consent to participate in the Maisha Fiti study. For this sub-study, 746 (74.4%) participants were HIV-negative and were included. The median age of these participants was 30 years (interquartile range (IQR) 24–37). Secondary education completion was low, with 67.5% having either no education or only completed primary education (Table 2). In their childhood, most participants reported experiencing an adverse family environment (89.4%), sexual and/or physical violence (78.4%), and witnessing war and/or community violence (91.3%). Overall, 63.2% had a current non-paying intimate partner and 29.4% solicited clients from public places such as the streets, buses or taxis where they were more likely to be vulnerable, with the others most commonly soliciting from locations such as bars or clubs. The median number of clients in the past seven days was 4 (IQR 1–6).

**Table 2:** Study sample characteristics and bi-variate associations with PrEP usage and adherence

	Total	PrEP use	PrEP adherence	
	HIV negative	Currently taking PrEP	Self-reported adherence	LFA adherence
	*N* = 746	*n* = 180/746 (24.1%)	*n* = 153/180 (85.0%)	*n* = 56/180 (31.1%)
**Level 1: Sociodemographic characteristics and individual-level factors**				
Current age				
Median (IQR)	30 (24–37)	30 (25–38)	30 (24–37)	32 (28–40)
18–24	200 (15.3%)	44 (22.0%)	40 (90.9%)	6 (13.6%)
25–34	286 (44.4%)	71 (24.8%)	62 (87.3%)	25 (35.2%)
35+	260 (40.3%)	65 (25.0%)	51 (78.5%)	25 (38.5%)
Education level				
None/some primary	117 (16.9%)	26 (22.2%)	24 (92.3%)	8 (30.8%)
Some secondary	380 (50.6%)	91 (23.9%)	79 (86.8%)	32 (35.2%)
Completed secondary/higher education	249 (32.5%)	63 (25.3%)	50 (79.4%)	16 (24.4%)
Adverse childhood experiences				
Ever lived on the street	91 (11.5%)	24 (26.4%)	20 (83.3%)	7 (29.2%)
Experienced adverse family/household environment	661 (89.4%)	160 (24.2%)	136 (81.3%)	48 (30.0%)
Experienced emotional violence	512 (69.2%)	127 (24.8%)	105 (82.7%)	44 (34.6%)
Experienced sexual and/or physical violence	582 (78.4%)	136 (23.4%)	115 (84.6%)	45 (33.1%)
Witnessed war and/or community violence	686 (91.3%)	166 (24.2%)	140 (84.3%)	49 (29.5%)
Wealth tertile				
Low	299 (39.2%)	80 (26.8%)	71 (88.8%)	18 (22.5%)
Middle	137 (18.0%)	35 (25.5%)	28 (80.0%)	7 (20%)
Upper	310 (42.7%)	65 (21.0%)	54 (83.1%)	31 (47.7%)
Mental health				
Moderate/severe depression and/or anxiety	180 (25.3%)	43 (23.9%)	36 (83.7%)	15 (34.9%)
Recent suicidal thoughts/behaviours	75 (10.2%)	15 (20.0%)	14 (93.3%)	7 (46.7%)
PTSD	102 (14.2%)	27 (26.5%)	25 (92.6%)	9 (33.3%)
Alcohol and/or substance use risk				
Low	400 (54.5%)	85 (21.3%)	74 (87.1%)	33 (38.8%)
Moderate	249 (33.0%)	64 (25.7%)	54 (84.4%)	19 (29.7%)
High	93 (12.5%)	30 (32.3%)	24 (80.0%)	4 (13.3%)
Internalised sex work stigma	533 (72.5%)	124 (23.3%)	103 (83.1%)	39 (31.5%)
Internalised PrEP stigma				
Feel ashamed of using PrEP	74 (10.4%)	15 (20.3%)	14 (93.3%)	4 (26.7%)
Feel embarrassed if others knew I was using PrEP	107 (14.9%)	21 (19.6%)	19 (90.5%)	6 (28.6%)
Feel empowered to use PrEP	533 (74.2%)	158 (29.6%)	136 (86.1%)	51 (32.3%)
Think I am not following community rules by using PrEP	271 (36.7%)	65 (24.0%)	56 (86.2%)	23 (41.1%)
**Level 2: Interpersonal- and community-level factors**				
Ever married	562 (78.5%)	141 (25.1%)	118 (83.7%)	49 (34.6%)
Is nulliparous (has no children)	81 (8.1%)	16 (19.8%)	11 (68.8%)	6 (37.5%)
Intimate partner (IP)				
Current non-paying IP	480 (63.2%)	116 (24.2%)	101 (87.1%)	36 (31.0%)
IP has knowledge of sex work	142 (19.8%)	41 (28.9%)	38 (92.7%)	15 (36.6%)
Knowledge of IP’s HIV-positive status	9 (1.2%)	4 (4.4%)	3 (75.0%)	0 (0.0%)
No experience of sexual and/or physical violence from IP in past 6 months	497 (66.3%)	127 (25.6%)	110 (86.6%)	40 (31.5%)
Sex work				
Age when first started selling sex				
Median (IQR)	21 (18–25)	20.5 (19–25)	20 (18–25)	23 (20–28)
≤ 15	51 (6.7%)	15 (29.4%)	14 (93.3%)	2 (13.3%)
16–17	83 (10.0%)	16 (19.3%)	14 (87.5%)	5 (31.3%)
18+	604 (83.3%)	147 (24.3%)	124 (84.4%)	49 (33.3%)
Place of selling sex				
Street/bus/taxis	212 (29.4%)	44 (20.8%)	35 (79.5%)	17 (38.6%)
Other	529 (70.7%)	136 (25.7%)	118 (86.8%)	39 (28.7%)
Number of clients in last 7 days				
Median (IQR)	4 (1–6)	4 (2–7)	4 (2–7)	5 (3–8)
0–5	436 (58.5%)	95 (21.8%)	78 (82.1%)	27 (28.4%)
5–9	201 (27.0%)	57 (28.4%)	51 (89.5%)	19 (33.3%)
10+	103 (14.5%)	28 (27.2%)	24 (23.3%)	10 (35.7%)
Anal sex with client in last 7 days	12 (1.6%)	4 (33.3%)	4 (100%)	0 (0.0%)
Sexual and/or physical violence from non-IP in last 6 months	402 (54.4%)	106 (26.4%)	92 (86.8%)	34 (32.1%)
Condom use at last sex	559 (75.8%)	143 (25.6%)	143 (100%)	45 (30.1%)
Experienced sex work stigma	510 (69.9%)	133 (26.1%)	113 (85.0%)	43 (32.3%)
Experienced PrEP stigma				
I will get a hard time if people know I am taking PrEP	278 (38.1%)	68 (24.5%)	59 (86.8%)	18 (26.5%)
People will judge me negatively if I am taking PrEP	312 (42.9%)	75 (24.0%)	66 (88.0%)	19 (25.3%)
I am at greater risk of violence or rape if taking PrEP	131 (18.1%)	28 (21.4%)	25 (89.3%)	9 (32.1%)
People will think I am acting responsibly if taking PrEP	273 (38.3%)	72 (26.4%)	65 (90.3%)	24 (33.3%)
Social support				
Does not have support from the community	120 (15.9%)	30 (25.0%)	25 (83.3%)	15 (50.0%)
Has support from sex worker organisations	85 (11.5%)	29 (34.1%)	27 (93.1%)	5 (17.2%)
Treated for sexually transmitted infection in last 30 days	166 (22.4%)	47 (28.3%)	39 (83.0%)	17 (36.2%)
**Level 3: Institutional- and structural-level factors**				
Skipped a meal in last 7 days due to financial constraints	233 (32.3%)	66 (28.3%)	55 (83.3%)	22 (33.3%)
Authorities				
Sexual and/or physical violence from authorities in past 6 months	83 (11.5%)	29 (34.9%)	25 (86.2%)	10 (34.5%)
Ever arrested for being a sex worker	394 (55.4%)	108 (27.4%)	91 (84.3%)	34 (31.5%)
Ever avoided arrest by having sex with police officer	96 (13.4%)	27 (28.1%)	25 (92.6%)	8 (29.6%)
Ever imprisoned for being a sex worker	102 (14.7%)	23 (22.5%)	19 (82.6%)	5 (21.7%)
Healthcare-related stigma				
Not avoiding seeking health services due to sex work stigma	633 (86.4%)	161 (25.4%)	136 (84.5%)	53 (32.9%)
Experienced discrimination in a health setting	19 (2.7%)	6 (31.6%)	6 (100%)	2 (33.3%)

### Current Usage of Oral-PrEP

Of the HIV-negative participants, 24.1% (95% CI = 0.21–0.27) self-reported taking oral-PrEP at baseline. In the multivariable hierarchical logistic regression (Table 3), after adjusting for all Level 1 variables as well as for age and clinic, sociodemographic and individual-level factors (Model 1), variables associated with an increased odds of self-reported adherence were having completed secondary education (aOR = 2.31; 95% CI = 1.20–4.43); feeling empowered to use PrEP (aOR = 5.12; 95% CI = 2.75–9.51); having a low or low-middle wealth tertile vs. upper-middle or high wealth (aOR = 1.72; 95% CI = 1.09–2.70); and a high alcohol and/or substance use score (aOR = 2.17; 95% CI = 1.23–3.83).

Interpersonal- and community-level factors (Model 2), after adjusting for all Level 2 variables, Model 1 variables as well as age and clinic, associated with an increased odds of self-reported adherence included having a current non-paying intimate partner (aOR = 1.63; 95% CI = 1.03–2.57); not having experienced sexual and/or physical violence from an intimate partner in the past six months (aOR = 1.72; 95% CI = 1.07–2.77); having support from sex worker organisations (aOR = 1.85; 95% CI = 1.02–3.37); and experiencing sex work stigma (aOR = 1.69; 95% CI = 1.05–2.71). Not having support from the community (aOR = 1.62; 95% CI = 0.96–3.11) trended towards increased self-reported current usage of oral-PrEP.

Institutional- and structural-level factors (Model 3), after adjusting for all Level 3 variables, Model 1 and 2 variables as well as age and clinic, associated with an increased odds of self-reported adherence included seeking health services in spite of sex work stigma (aOR = 2.60; 95% CI = 1.28–5.27) and experiencing sexual and/or physical violence from authorities in the past six months (aOR = 1.77; 95% CI = 1.0-3.14).

**Table 3:** Multivariable logistic regression: associations with current usage of oral-PrEP (of all HIV negative)

	Crude odds ratio (95% CI)	Adjusted odds ratio (95% CI)	*p*-value
Model 1*			
Education level			
None/some primary	Reference	Reference	
Some secondary	1.06 (0.65–1.73)	1.40 (0.77–2.59)	
Completed secondary/higher education	1.27 (0.76–2.12)	2.31 (1.20–4.43)	**0.02**
Adverse childhood experiences			
Experienced emotional violence	1.18 (0.82–1.70)	1.22 (0.79–1.89)	0.36
Wealth tertile			
Low/low-middle	1.29 (0.89–1.87)	1.72 (1.09–2.70)	
Middle	1.17 (0.73–1.87)	1.50 (0.87–2.58)	
Upper middle/upper	Reference	Reference	**0.05**
Alcohol and/or substance use risk			
Low	Reference	Reference	
Moderate	1.20 (0.83–1.73)	1.32 (0.85–2.05)	
High	1.74 (1.06–2.85)	2.17 (1.23–3.83)	**0.03**
Feel empowered to use PrEP	3.92 (2.35–6.54)	5.12 (2.75–9.51)	**< 0.001**
Model 2**			
Is nulliparous (has no children)	0.76 (0.42–1.37)	1.19 (0.56–2.52)	0.66
Current non-paying IP	1.04 (0.73–1.47)	1.63 (1.03–2.57)	**0.04**
No experience of sexual and/or physical violence from IP in past 6 months	1.25 (0.87–1.79)	1.72 (1.07–2.77)	**0.03**
Experienced sex work stigma	1.47 (1.00-2.14)	1.69 (1.05–2.71)	**0.03**
Social support			
Does not have support from the community	1.20 (0.77–1.87)	1.62 (0.92–2.87)	**0.09**
Has support from sex worker organisations	1.91 (1.19–3.09)	1.85 (1.02–3.37)	**0.04**
Model 3***			
Skipped a meal in last 7 days due to financial constraints	1.35 (0.95–1.92)	1.36 (0.88–2.10)	0.17
Sexual and/or physical violence from authorities in past 6 months	1.91 (1.18–3.09)	1.77 (1.0-3.14)	**0.05**
Healthcare-related stigma			
Seeking health services despite sex work stigma	1.66 (0.97–2.85)	2.60 (1.28–5.27)	**0.01**
Experienced discrimination in a health setting	1.48 (0.56–3.90)	1.63 (0.88–2.10)	0.47

*Model 1 adjusted for all Level 1 variables (education level, adverse childhood experiences, wealth tertile, mental health problems, alcohol and substance use risk, and internalised sex work and pre-exposure prophylaxis (PrEP) stigma), age and clinic.

**Model 2 adjusted for Level 1 variables with *p* < 0.1, all Level 2 variables (marital status, children, intimate partners, sex work location and clients and condom use, and experienced sex work and PrEP stigma), age and clinic.

***Model 3 adjusted for Level 1 and 2 variables with *p* < 0.1, all Level 3 variables (hunger, institutional violence, and healthcare-related stigma), age and clinic.

### Comparison of Self-reported Oral-PrEP Adherence Relative to Lateral Flow Assay Oral-PrEP Adherence

**Fig. 2:**
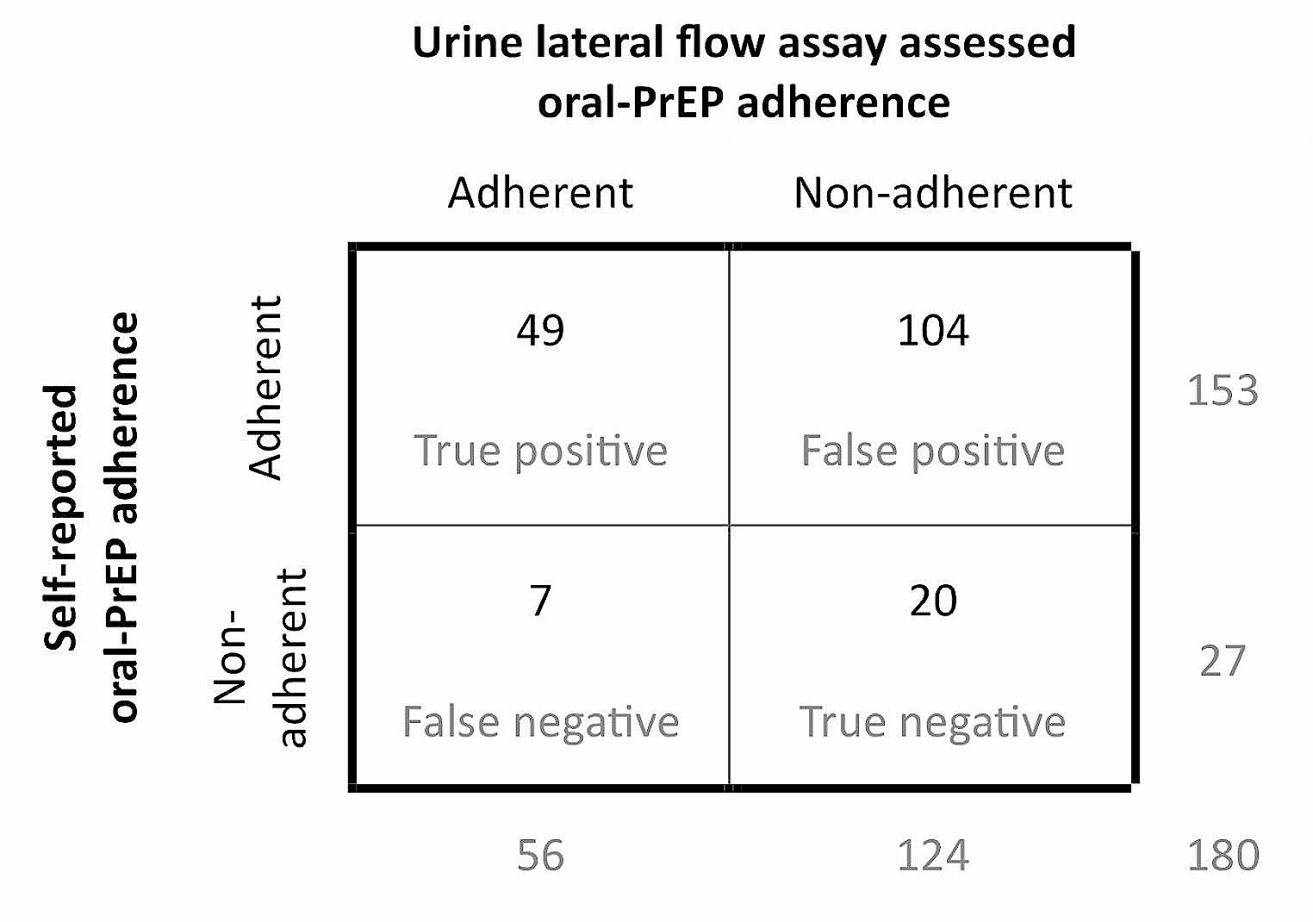
Test characteristics of lateral flow assay

In ROC analyses to evaluate how well self-reported adherence over the past seven days predicted LFA-assessed adherence to oral-PrEP, the area under the ROC curve was 0.52 (95% CI 0.46–0.57), suggesting that self-report performed poorly in predicting adherence assessed by LFA. The Kappa statistic was 0.02, indicating very poor intermeasure agreement between self-report and LFA-assessed adherence. The accuracy of self-reported adherence compared to LFA-assessed adherence was estimated to be 38.33% (95% CI = 31.20-45.86%). As shown in Fig. 2, among the 124 FSWs who were non-adherent as assessed by LFA, 20 were also non-adherent by self-report, indicating 16.1% specificity (95% CI = 10.8-21.5%). Of the 56 women adherent as assessed by LFA, 49 were adherent by self-report, indicating 87.5% sensitivity (95% CI = 82.7-92.3%).

### Associations with Urine Assay-measured Adherence

Of the participants who self-reported adherence of oral-PrEP, 85.0% (95% CI = 0.79–0.90) reported adherence over the last seven days, while 31.1% (95% CI = 0.24–0.38) were shown to have adhered to oral-PrEP in the last four to seven days using the LFA measure. In the multivariable analyses (Table 4), after adjusting for all Level 1 variables as well as age and clinic, sociodemographic and individual-level factors (Model 1), factors associated with an increased odds of LFA-measured adherence included having completed primary education or less (aOR = 3.68; 95% CI = 1.45–9.31); having an upper-middle or high wealth tertile as opposed to low or low-middle (aOR = 5.27; 95% CI = 2.04–13.59); and having experienced emotional violence in childhood (aOR = 2.51; 95% CI = 1.05–6.03).

The only interpersonal- and community-level factor (Model 2), after adjusting for all Level 2 variables and Model 1 variables, as well as age and clinic, associated with increased LFA-measured adherence was being nulliparous (aOR 4.75; 95% CI = 1.29–17.49), whereas in Model 3 (institutional- and structural-level factors), after adjusting for all Level 3 variables, Model 1 and 2 variables, as well as age and clinic, having skipped a meal in the past seven days due to financial constraints (aOR = 2.31; 95% CI = 0.92–5.77) trended towards increased LFA-measured adherence.

**Table 4:** Multivariable logistic regression: associations with lateral flow assay PrEP adherence (of those with current PrEP usage)

	Crude odds ratio (95% CI)	Adjusted odds ratio (95% CI)	*P*-value
Model 1*			
Education level			
None/some primary	1.34 (0.50–3.63)	3.48 (1.04–11.59)	
Some secondary	1.77 (0.87–3.60)	3.68 (1.45–9.31)	
Completed secondary/higher education	Reference	Reference	**0.02**
Adverse childhood experiences			
Experienced emotional violence	1.66 (0.80–3.48)	2.51 (1.05–6.03)	**0.04**
Wealth tertile			
Low/low-middle	Reference	Reference	
Middle	0.90 (0.34–2.41)	1.14 (0.35–3.71)	
Upper middle/upper	3.01 (1.48–6.16)	5.27 (2.04–13.59)	**0.002**
Alcohol and/or substance use risk			
Low	4.15 (1.20-14.38)	4.85 (1.07–21.99)	
Moderate	3.96 (1.05–14.93)	2.91 (0.63–13.53)	
High	Reference	Reference	0.10
Feel empowered to use PrEP	1.15 (0.40–3.34)	0.70 (0.21–2.31)	0.56
Model 2**			
Is nulliparous (has no children)	1.90 (0.64–5.59)	4.75 (1.29–17.49)	**0.02**
Current non-paying IP	1.08 (0.56–2.09)	1.74 (0.57–5.34)	0.33
No experience of sexual and/or physical violence from IP in past 6 months	0.99 (0.50–1.97)	1.35 (0.50–3.64)	0.56
Experienced sex work stigma	1.06 (0.51–2.21)	0.86 (0.34–2.17)	0.75
Social support			
Does not have support from the community	2.55 (1.15–5.65)	2.15 (0.8205.63)	0.12
Has support from sex worker organisations	0.39 (0.14–1.07)	0.37 (0.09–1.45)	0.15
Model 3***			
Skipped a meal in past 7 days due to financial constraints	1.16 (0.61–2.21)	2.31 (0.92–5.77)	**0.07**
Sexual and/or physical violence from authorities in past 6 months	1.20 (0.52–2.76)	1.15 (0.37–3.51)	0.81
Healthcare-related stigma			
Seeking health services despite sex work stigma	2.76 (0.75–10.17)	3.91 (0.56–27.53)	0.17
Experienced discrimination in a health setting	1.14 (0.21–6.25)	6.05 (0.62–59.41)	0.12

*Model 1 adjusted for all Level 1 variables (education level, adverse childhood experiences, wealth tertile, mental health problems, alcohol and substance use risk, and internalised sex work and pre-exposure prophylaxis (PrEP) stigma), age and clinic.

**Model 2 adjusted for Level 1 variables with *p* < 0.1, all Level 2 variables (marital status, children, intimate partners, sex work location and clients and condom use, and experienced sex work and PrEP stigma), age and clinic.

***Model 3 adjusted for Level 1 and 2 variables with *p* < 0.1, all Level 3 variables (hunger, institutional violence, and healthcare-related stigma), age and clinic.

## Discussion

In Nairobi, Kenya, despite the free oral-PrEP provision by the government, fewer than a quarter of the FSWs that were HIV-negative self-reported currently taking oral-PrEP. Of these, the majority self-reported oral-PrEP adherence over the past seven days, yet only a third were actually adherent to oral-PrEP based on a highly specific, objective urine LFA metric. Self-reported adherence in the past seven days was poorly associated with LFA-assessed adherence. Our study highlights the limitations of self-reported measures and, using objective adherence assessment, the individual, interpersonal, and structural facilitators and barriers to oral-PrEP usage.

Only the completion of no more than primary school education was significantly associated with both self-reported and LFA-assessed adherence. Other factors associated with self-reported adherence included feeling empowered to use oral-PrEP, having a lower wealth tertile, a higher alcohol and/or substance use risk score, having a current intimate partner, no recent experience of intimate partner violence, experiencing sex work stigma, lack of community support, having support from sex worker organisations, and seeking healthcare despite sex work stigma. Higher LFA-measured adherence was associated with having a higher wealth tertile, experiencing emotional violence in childhood, and being nulliparous.

Overall, our results are consistent with studies among FSWs globally, which report high levels of interest in oral-PrEP but low levels of uptake and adherence. Levels of self-reported oral-PrEP initiation in our population were similar to routine programmatic data collected from a national study involving almost 64,000 PrEP-eligible FSWs in Kenya, of whom less than a third initiated oral-PrEP [62]. PrEP adherence is important to optimise efficacy, and with adequate training and sensitisation, the urine-based tenofovir (TFV) LFA could be beneficial as a real-time tool to facilitate discussions about PrEP adherence. A feasibility study in Kenya exploring reactions to the LFA test found that both women using oral-PrEP and healthcare professionals felt that the test would be acceptable for use in clinical settings, provided clients were clearly informed of the procedure and healthcare professionals were supportive and non-judgemental. In this setting, the LFA test could be used to initiate discussion about prevention-effective adherence, challenges related to adherence, and the availability of different HIV prevention options [63].

Our results showed that a lower level of education was associated with both objective and self-reported adherence. Fewer years of education is a risk factor for HIV risk behaviours [64], and it could be that the perception of having a higher risk of HIV acquisition due to their socioeconomic circumstances motivated FSWs with lower levels of education to initiate and adhere to oral-PrEP. Experiencing emotional violence in childhood was associated with higher objectively measured adherence in our study. FSWs who experienced neglect or emotional abuse in childhood may have developed coping mechanisms to deal with stress and adversity, and as such the higher level of adherence to PrEP may be viewed as a proactive coping strategy to take control of their health. As these FSWs were participants in a study, this pattern of behaviour may also stem from a desire to gain approval from staff as well as a desire to take control of their health.

In Kenya, oral-PrEP has been successfully rolled out free of charge, both geographically and by priority population. Financial insecurity is a driver of sexual risk behaviours [1], and the provision of PrEP at no direct cost to FSWs themselves may explain why in our study, being in a lower wealth tertile was associated with increased odds of self-reported adherence. However, being in a higher wealth tertile was associated with increased odds of LFA-assessed adherence. Adhering to oral-PrEP requires FSWs to bear recurring costs of transportation and time during which they could be earning money [65], making it difficult for those not in a higher socioeconomic position to commit to a daily oral-PrEP regimen.

In our study, feeling empowered to use PrEP, having a current non-paying intimate partner, and being in a non-violent relationship increased the odds of self-reported oral-PrEP adherence. Previous qualitative research has uncovered how power differentials pose a challenge to women’s PrEP usage [66]. On an individual level, PrEP use can be an empowering, female-controlled means of self-protection and improved health and well-being [67, 68]. A study among FSWs in Uganda found that being able to charge a higher price for condomless sex while not risking HIV was a form of empowerment as it made business sense [69]. Women in non-violent relationships may have higher perceived control and thus reduced HIV risk behaviours, contributing to increased oral-PrEP current use [70, 71]. FSWs in our study may have taken up oral-PrEP for these reasons, but having a non-paying intimate partner may have negatively impacted adherence. This could be because intimate partners may mistake the daily oral-PrEP pills for antiretrovirals, thinking that the FSW is HIV-positive and has been untruthful about their diagnosis [16, 72]. It could also create conflict by raising questions about fidelity and trust [73]. We also reported that having no children was associated with increased LFA-measured adherence, but this was only reported by six FSWs. Research on this is scarce, but one potential reason may be that with fewer responsibilities, FSWs could have more flexibility to prioritise their health.

A recent systematic review and meta-analysis of alcohol use among FSWs in low- and middle-income countries found that over a third of FSWs in SSA reported harmful alcohol use in the context of sex work [74]. Some studies have shown that alcohol use is associated with lower levels of adherence to oral-PrEP [67, 75]. Our study reflects these results, with low alcohol and/or substance use trending towards being associated with increased LFA-assessed adherence. Conversely, in our study, high alcohol and/or substance use was associated with increased odds of self-reported adherence. Qualitative studies have described how alcohol use increased FSWs’ perceived need for oral-PrEP due to concerns about condom negotiation and increased likelihood of sexual violence [15]. This may explain how FSWs with harmful alcohol/substance use in our study perceived themselves to be at higher risk of HIV acquisition [1], and therefore higher self-reported adherence, despite challenges with daily adherence.

In our study, seeking health services despite stigma related to sex work was associated with increased self-reported oral-PrEP adherence, but not with LFA-assessed adherence. Whilst much PrEP education to date has focused on providing knowledge to FSWs, the focus should shift to include healthcare workers to reduce stigma. Oral-PrEP providers should receive sensitivity training to address healthcare worker discrimination, as stigmatising attitudes and moral beliefs may create tension in interactions with FSWs [19, 76]. FSWs experience stigma as a result of their work, and accessing oral-PrEP may increase that stigma as others may perceive them as either having a high risk of HIV or being mistaken for living with HIV as a result of taking daily antiretroviral medication [18, 67].

Improving the levels of oral-PrEP uptake and adherence among FSWs would help to achieve global HIV prevention goals. To achieve this, oral-PrEP service delivery will need to meet the needs of the population [77]. Discussions regarding PrEP usage in FSWs should include and be centred around FSWs, while supporting and empowering them to recognise their risk and control their regimen. Sex Worker Advocacy Groups in Kenya could collaborate with Ministry of Health officials and local media to effectively increase PrEP awareness in key populations and reduce stigma and misinformation. Oral-PrEP delivery programs in Kenya are predominantly clinic-based, and FSWs often work at night-time or irregular hours [76]. FSWs may also be more comfortable with their peers providing information, services and referrals as they better understand the context and risks of sex work [15, 78]. As such, expanded and/or drop-in clinic hours, integration of PrEP services with other health services (including contraception, obstetric care, primary and mental healthcare, and harm reduction services), and training mobile peer outreach workers could increase accessibility to HIV prevention services and therefore improve oral-PrEP usage among this key population. Recent advances in long-acting PrEP methods such as the dapivirine ring and injectable cabotegravir may also provide FSWs with acceptable, discrete, and convenient options [79–81].

This study is one of the first in sub-Saharan Africa to quantitatively evaluate factors associated with oral-PrEP usage specifically among self-identified FSWs. A key strength of this study is the use of a novel, real-time urine tenofovir test to objectively measure oral-PrEP adherence. Participants provided consent for biological samples to be frozen for later testing of antiretrovirals regardless of HIV status, and social desirability bias was minimised as oral-PrEP usage was not a primary aim of the study. Important limitations of this study include the cross-sectional design, meaning causality cannot be inferred and trends in PrEP usage over time were not available. Pill count data was not collected as part of the Maisha Fiti study, and so we could not use that as an additional comparator to self-reported adherence. The urine LFA test can accurately detect the presence of tenofovir over the past four to seven days, and thus can only measure short-term adherence. The results of this study will not be representative of all FSWs in Nairobi, as those not registered at a SWOP clinic would not have been identified for inclusion in the study.

## Conclusions

This study provides valuable insights into FSWs’ self-reported and LFA-measured adherence to oral-PrEP in Nairobi. Unfortunately, oral-PrEP adherence was not optimal for effective HIV prevention given the high HIV incidence among FSWs in Nairobi. The LFA test is an affordable, real-time measure of PrEP dosing that warrants further research regarding its role in improving PrEP usage among key populations. Interventions to improve oral-PrEP adherence in this population should involve both FSWs and healthcare providers and focus on aspects such as combination prevention, addressing stigma, collaborative information sharing, empowerment, counselling, and reduction of alcohol and substance use. Newer PrEP delivery methods, such as long-acting dosages may provide an alternative for those who find daily oral-PrEP challenging.
